# CCAT1/FABP5 promotes tumour progression through mediating fatty acid metabolism and stabilizing PI3K/AKT/mTOR signalling in lung adenocarcinoma

**DOI:** 10.1111/jcmm.16815

**Published:** 2021-08-25

**Authors:** Jing Chen, Yaser Alduais, Kai Zhang, Xiaoli Zhu, Baoan Chen

**Affiliations:** ^1^ Department of Hematology and Oncology School of Medicine Zhongda Hospital Southeast University Nanjing China; ^2^ Department of Respiratory Medicine Nanjing First Hospital Nanjing China; ^3^ Department of Respiratory Medicine School of Medicine Zhongda Hospital Southeast University Nanjing China

**Keywords:** angiogenesis, CCAT1, fatty acid metabolism, lung adenocarcinoma, ubiquitination

## Abstract

Long non‐coding RNA (lncRNA) colon cancer associated transcript 1 (CCAT1) has been identified as an oncogene in many cancers, but its role in lung adenocarcinoma (LUAD) remains to be further investigated. We identified the upregulation of CCAT1 in LUAD tissues and LUAD cells. Through RNA pull‐down and mass spectrometry analysis, we obtained the interacting proteins with CCAT1 and discovered their functional relation with ‘signal transduction’, ‘energy pathways’ and ‘metabolism’ and revealed the potential of CCAT1 on fatty acid (FA) metabolism. For mechanism exploration, we uncovered the mediation of CCAT1 on the translocation of fatty acid binding protein 5 (FABP5) into nucleus by confirming their interaction and localization. Also, CCAT1 was discovered to promote the formation of the transcription complex by RXR and PPARγ so as to activate the transcription of CD36, PDK1 and VEGFA. Moreover, we found that CCAT1 regulated the activity of AKT by promoting the ubiquitination of FKBP51 through binding with USP49. Subsequently, cell function assays revealed the enhancement of CCAT1 on LUAD cell proliferation and angiogenesis in vitro and in vivo. Collectively, CCAT1 regulated cell proliferation and angiogenesis through regulating FA metabolism in LUAD, providing a novel target for LUAD treatment.

## 1 INTRODUCTION

Lung adenocarcinoma (LUAD) is the commonest subtype of non‐small cell lung cancer (NSCLC).^1^, ^2^ Despite significant progress achieved in clinical and experimental ontology, the prognosis of LUAD still presents a gloomy prospective.^3^, ^4^ Early diagnosis is important for the successful treatment of LUAD, and however, patients with LUAD usually are diagnosed at advanced stage or with metastasis.^5^ Therefore, the identification of novel diagnosis and therapeutic biomarkers for LUAD patients is in great urgency.

Over the past decade, with development of whole‐genome sequencing technology, particular attention has been paid on the exploding class of transcripts of long non‐coding RNAs (lncRNAs), arbitrarily defined according to a length over 200 nucleotides.^6^, ^7^ Once considered as transcriptional noise, lncRNAs are now revealed by steadily growing lists to play authentic biological roles. Many lncRNAs are identified to share common characteristics with protein‐coding transcripts, such as RNA polymerase II mediated transcription, canonical splice site motifs based splicing and frequent 3’‐end poly‐adenylation.^8^ Functional characterization has shown that lncRNAs exhibit various biological functions such as transcriptional regulation, titration of miRNAs or proteins, and bridging of proteins or chromatin regions.^9^ To date, although only a fraction of functional lncRNAs have been well characterized, their participation at every level of the multi‐level regulated gene expression pathway has been revealed.^10^ For instance, lncRNAs have been implicated to modulate gene expression through controlling protein synthesis, RNA maturation and transport, and transcriptionally silencing gene via regulating the chromatin structure.^11^ Through different regulatory mechanisms, lncRNAs are involved in the regulation on numerous biological processes, including cell cycle, proliferation, differentiation and apoptosis.^12^ Accordingly, profiling of lncRNAs has led us to the discovery of their abnormal expression pattern in various types of cancer,^13^ indicating the potential value of lncRNAs as cancer biomarkers or therapeutic targets. Despite these advances, most lncRNAs remain partially uncharacterized, especially in lung adenocarcinoma.

LncRNAs, as key transcriptional regulators of central metabolic pathways, can provide proliferating advantages through significantly enhancing the biosynthesis of FA. And several investigations have elucidated the interaction between fatty acid (FA) metabolism and lncRNA. For instance, lncRNA LNMICC contributes to the nodal metastasis of cervical cancer through reprogramming FA metabolism.^14^ However, it remains largely unknown how lncRNAs cope with the reprogramming of FA metabolism in lung adenocarcinoma cells, thus resulting in the progression of lung adenocarcinoma.

Colon cancer‐associated transcript‐1 (CCAT1), firstly identified by Nissan et al., was highly expressed in colorectal cancer.^15^ Recently, the oncogenic property and regulatory mechanism of CCAT1 has been reported in various types of cancer, including squamous cancer^16^ and oesophageal squamous cell carcinoma.^17^ However, the biological functions and regulatory mechanism of CCAT1 in the control of LUAD tumorigenesis and the association with FA metabolism have not been well elucidated.

In our present study, we found CCAT1 was significantly upregulated in LUAD cell lines and identified its correlation with FA metabolism. Also, CCAT1 was revealed to drive the transcription complex formed by RXR and PPARγ therefore activated the transcription of CD36, PDK1 and VEGFA. Moreover, we uncovered the regulation of CCAT1 on the activity of AKT by promoting the ubiquitination of FKBP51 through the interaction with USP49. Gain‐ and loss‐of‐function assays revealed the oncogenic effect of CCAT1 on LUAD cell proliferation and angiogenesis. Finally, in vivo assays confirmed the results of in vitro assays. In conclusion, current study revealed the role of CCAT1 on the FA metabolism in lung adenocarcinoma, which provided a novel therapeutic target for LUAD patients.

## 2 MATERIALS AND METHODS

### 2.1 Tissue collection

Ten lung cancer tissues and paired non‐cancerous tissues were dissected from patients in Nanjing First Hospital. The tissues were stored under −80℃ after dissection for subsequent use. All patients who underwent the surgical removal of tumours had not received any other radio‐ or chemo‐therapy and had signed the written informed consent. Assays on tissues were approved by the ethic committee of Nanjing First Hospital.

### 2.2 Cell lines and cell culture

Two human non‐small‐cell lung cancer cell lines (SPC‐A1, A549 and H1299) were bought from the Cell Bank of Typical Preservation Committee, Chinese Academy of Science. And HUVECs were purchased from ATCC (American Type Culture Collection, Manassas, VA, USA). SPC‐A1 and A549 cells were cultured at 37℃ in the RPIM1640 (Gbico, Thermo Fisher Scientific, Waltham, MA, USA) suffused by 10% of foetal bovine serum (FBS; Gbico, Thermo Fisher Scientific), 100 U m/L penicillin and streptomycin (Mediatech Inc., Manassas, VA, USA) under a humid air with 5% CO_2_. HUVECs were cultured in complete Growth Medium F12K+0.03–0.05 mg/ml ECGS+0.1 mg/ml Heparin+10% FBS+P/S under a humid air with 5% CO_2_.

### 2.3 RNA isolation and quantitative reverse transcriptase‐polymerase chain reaction (qRT‐PCR)

A total amount of RNA was isolated from cells applying TRIzol reagent following the standard protocols. The reverse transcription of total RNA was performed by Revert Aid First Strand cDNA Synthesis Kit followed by quantification using spectrophotometry. The Maxima SYBR Green/ROX qPCR Master Mix was employed for real‐time polymerase chain reaction assay with GAPDH or U1 used as the internal control.

### 2.4 Cell transfection

Specific si‐RNAs targeting CCAT1 was applied to silence CCAT1 whereas pcDNA3.1 with CCAT1 sequence was used to overexpress CCAT1. siCtrl and pcDNA3.1 empty vectors served as negative controls. All of the plasmid vectors above were acquired from Shanghai Integrated Biotech Solutions Co., Ltd. (Shanghai, China). Transfection of these vectors into cells as demand was carried out with the use of Lipofectamine 2000 or siRNA mate (Invitrogen).

### 2.5 Pull‐down, mass spectrometry and RNA immunoprecipitation assays

For the RNA pull‐down assay, Magnetic RNA‐Protein Pull‐Down kit (Pierce, MA, USA) was applied referring to the manufacturer's directions. Protein bands were then silver‐stained. Bands of which repeatedly enriched on the biotin‐labelled probe with CCAT1 rather than antisense CCAT1 were analysed by mass spectrometry (MS) and validated using western blotting. For the RNA immunoprecipitation (RIP), Magna RIP RNA‐Binding Protein Immunoprecipitation Kit (Millipore, MA, USA) was employed following the manufacturer's guide.

### 2.6 Chromatin immunoprecipitation (ChIP)

For the ChIP assay, EZ‐Magna ChIP TMA Chromatin Immunoprecipitation Kit (Millipore, Billerica, MA, USA) was used according to the manufacturer's protocols. The formaldehyde cross‐linked chromatin from cells was sonicated for the generation of DNA fragments between 200 to 300 bp. Antibodies against PPAR and RXR (Abcam) were used to precipitate the chromatin DNA. The quantification of the enrichment of PDK1, VEGFA and CD36 promoter were performed by qRT‐PCR. Anti‐IgG served as control.

### 2.7 CoIP

For the CoIP assay, total cells were lysed 30 min with cell lysis buffer under 4℃. Then, the lysate was incubated with the antibodies against FKBP51 and UBI overnight. After adding protein G sepharose agitate, the sample 4℃ for 2 h. Following washing, the immunoprecipitants were separated using SDS‐PAGE and analysed via immunoblotting together with indicated antibodies.

### 2.8 Luciferase reporter assay

The luciferase reporter vector with wild type or mutant PDK1, VEGFA and CD36 promoter were transfected with pcDNA3.1/CCAT1 or empty pcDNA3.1 vector into cells using Lipofectamine 2000 reagent (Invitrogen) under the manufacturer's directions. 48 h after transfection, the luciferase activity was analysed by the Dual Luciferase Reporter Gene Assay Kit (Beyotime). Relative luciferase activities were normalized to Renilla luciferase activity.

### 2.9 Immunofluorescence (IF)

4% of formaldehyde was used to fix the cells for 15 min. Then, pepsin treated cells were dehydrated the using ethanol and permeabilized for 20 min by Triton X‐100 (Sigma‐Aldrich). After the blocking was performed with goat serum, incubate the cells overnight with antibodies against FABP5 under 4℃. Following washing of the primary antibodies, incubate cells for 1 h with rhodamine‐conjugated secondary antibody. For nuclear staining, the washed cells were incubated with DAPI (Invitrogen). The cells were observed under a confocal microscopy.

### 2.10 In situ hybridization (ISH)

The ISH probes were generated by Exiqon (Shanghai, China). ISH Kit (Boster Bio‐Engineering Company, Wuhan, China) was used to carry out ISH. The stained tissues were observed and then scored respectively by two pathologists who were blinded to the clinical parameters.

### 2.11 Protein Extraction, Western blotting and antibodies

For western blot assay, cell lysates were added with RIPA buffer with protease and phosphatase inhibitor (Thermo Fisher Scientific, Houston, TX). Following the centrifuging at 12,000 *g* under 4℃ for 12 min, the supernatants were examined by Western blotting. Antibodies used were as follows: anti‐FABP5 (Cat#:ab37267, abcam, UK), anti‐PDK1 (Cat#:ab52893, abcam, UK), anti‐p‐AKT (T308, Cat#:13038, Cell Signaling Technology), anti‐p‐AKT (Ser473, Cat#:4060S, Cell Signaling Technology), anti‐AKT (Cat#:4691, Cell Signaling Technology), anti‐p‐mTOR (Cat#:ab109268, abcam, UK), anti‐mTOR (Cat#:ab32028, abcam, UK), anti‐ACC1 (Cat#:ab45174), anti‐CD36 (Cat#:ab133625), anti‐VEGFA (Cat#:ab37267, Abcam, UK), anti‐ACOX1 (Cat#:ab184032, Abcam, UK) and anti‐FASN (Cat#:ab128870, Abcam, UK), anti‐USP49 (Proteintech), anti‐FKBP51 (Cat#:ab126715, Abcam, UK) and anti‐GAPDH from (Santa Cruz Biotechnology; Cambridge, MA).

### 2.12 Subcellular fractionation

PARIS Kit (Invitrogen) was used to separate the nuclear and cytosolic fractions under the manufacturer's guides. RNAs in cytoplasm and nuclei were isolated and then extracted from cells, and the levels of CCAT1 and FABP5 in the cytoplasm and nuclei were detected using qRT‐PCR or WB. TERC, Histone3 and GAPDH were examined as fractionation indicators.

### 2.13 EdU Incorporation Assay

Total cells were plated onto 96‐well cell culture plates with a concentration of 1 × 10^4^ cells per well. After transfection, 10 μmol/L of EdU (Roche Diagnostics, Mannheim, Germany) was added 24 h before the termination of the incubation of test reagent and integrated into the proliferating cells. Then, the fixed cells were neutralized for 10 min by glycine (2 mg/ml). Following washing, incubate the cells for 30 min with PBS supplemented with 0.1% Triton X‐100 (PBST). Later, the washed cells were labelled by 5 μg/ml of DAPI at 37℃ for 1 h. The EdU‐positive cells were presented as a percentage of control level.

### 2.14 Quantification of neutral lipids

The lipophilic fluorescence dye BODIPY 493/503 (Invitrogen) was employed for monitoring the neutral lipid accumulation in A549 cells.

### 2.15 Tube formation

For the tube formation assay, each well of the 96‐well plate was added with 50 μl of Matrigel (BD Biosciences, San Jose, CA, USA). Following the polymerizing for 30 min at 37℃, the harvested HUVECs from co‐culture system were seeded into the Matrigel with a concentration of 3 × 10^4^ cells/well. After 6 h incubation in a humid incubator with 5% CO_2_ at 37℃, the tubules were observed using an IX71 inverted microscope.

### 2.16 Animal models

Animal assays had obtained the approval of the Ethics Committee of Nanjing First Hospital. BALB/C nude mice aged 6–8 weeks were injected with luciferase A549 cells with lv‐CCAT1 or lv‐vector transfection for the generation of the xenografts. After 4 weeks, the tumours were dissected from mice and subjected to the observation of metastasis and HE staining. The tumour volume was examined referring to the equation as follows: volume = length × width^2^ × 0.5.

### 2.17 Immunohistochemistry (IHC)

For Immunohistochemistry assay, after the deparaffinizing the slides, the antigen retrieval was conducted in a steam cooker with 1 mM EDTA for 1.5 min. Antibody against FABP5 (Cell Signaling Technology, Danvers, MA, USA) was added and subjected to overnight incubation at 4℃. And the secondary antibody was added at room temperature for 15 min. 3‐amino‐9‐ethylcarbazole or diaminobenzidine served as chromogens. Counterstain the slides using haematoxylin before mounting.

### 2.18 Statistical analysis

The experiments were repeated three times, and the resulting data are presented after statistical processing. Data from multiple independent experiments are expressed as mean ± standard deviation. One‐way analysis of variance followed by Dunnett's or Tukey's multiple comparison test was performed to determine the significance of differences among groups. Statistical analysis was carried out on SPSS 13.0 software (SPSS; North Chicago, IL, USA). Differences were considered statistically significant at *p* < 0.05 in all experiments. *p < 0.05, **p < 0.01, ***p < 0.001.

## 3 RESULTS

### 3.1 Screened CCAT1‐associated RNA‐binding proteins (RBPs) involved in lung adenocarcinoma cells

**FIGURE 1:**
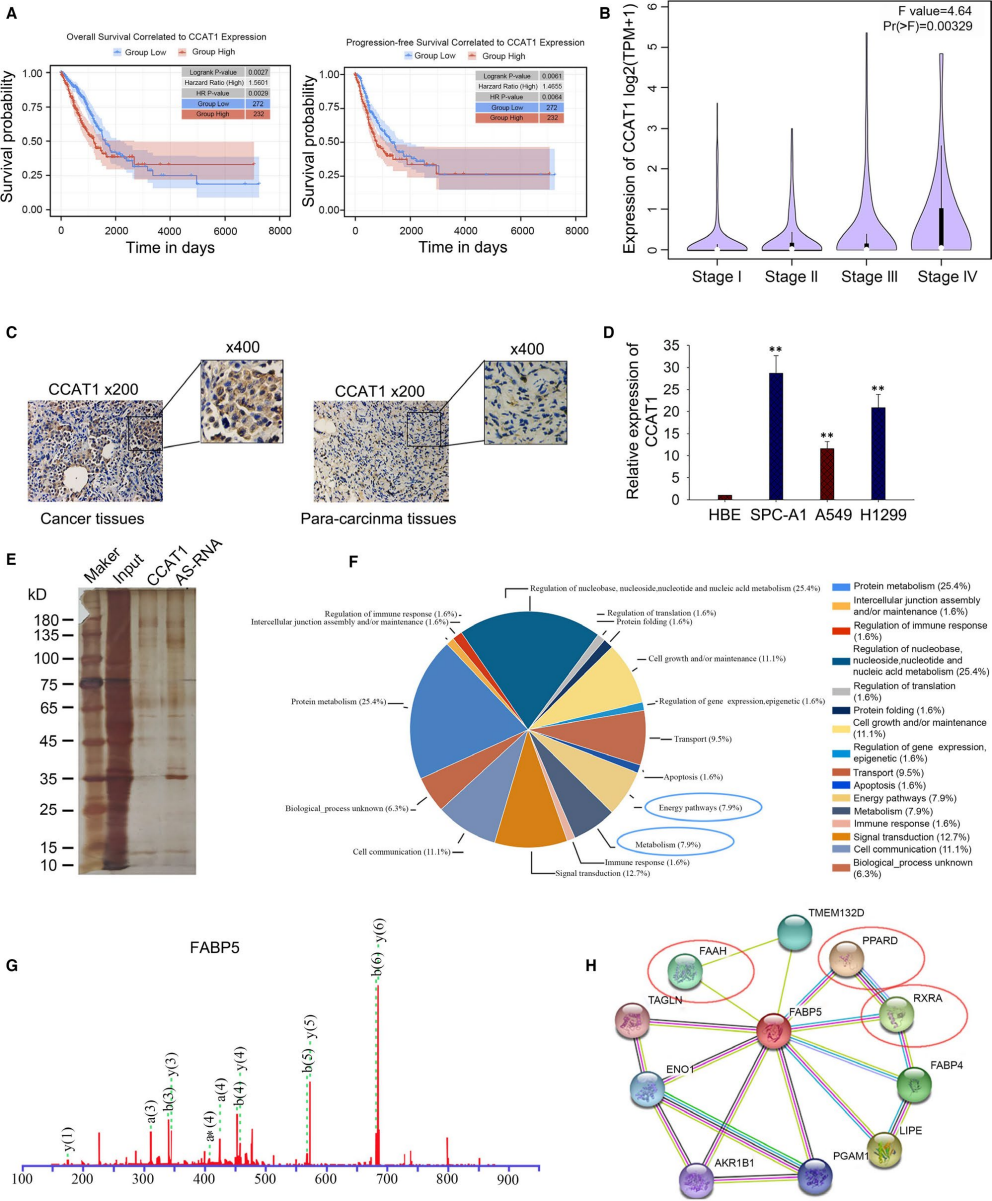
Screened CCAT1‐associated RBPs involved in LUAD cells (A) Association between CCAT1 expression and LUAD prognosis was analysed by Kaplan‐Meier analysis based on TCGA data. (B) qPCR results showed that high level of CCAT1 was related to advantaged stage of LUAD. (C) ISH presented a higher expression of CCAT1 in LUAD tissues. (D) qPCR was used to detect CCAT1 level in LUAD cell lines. Three biological replicates each group. Results analysed by one‐way ANOVA. (E) Pull‐down assay was used to find the binding partners with CCAT1. (F) GO analysis was used to analyse the coding genes of interacting proteins with CCAT1. (G) The mass spectrum peak of fatty acid binding protein 5 (FABP5). (H) The protein‐protein interaction analysis identified PPAR and RXR as metabolism‐related proteins interacting with FABP5. FAAH, fatty acid amide hydrolase; TMEM132D, transmembrane protein 132D; PPARD, peroxisome proliferator activated receptor delta; RXRA, retinoid X receptor alpha; FABP4, fatty acid binding protein 4; LIPE, lipase E; PGAM1, phosphoglycerate mutase 1; AKR1B1, Aldo‐Keto reductase family 1 member B; ENO1, Enolase 1; TAGLN, Transgelin

To probe the biological role of CCAT1 in LUAD, we first analysed the association between CCAT1 expression and the survival rate of LUAD patients from TCGA‐PANCANCER data. As shown in Figure 1A, high level of CCAT1 was negatively correlated with the overall survival (OS) (Logrank *p* = 0.0027, *n* (high) = 232, *n* (low) = 272) and disease free survival (DFS) (Logrank *p* = 0.0061, *n* (high) = 232, *n* (low) = 272). And we found that high level of CCAT1 was associated with advanced stage (F value = 4.64, Pr(>F) = 0.00329, Figure 1B). ISH results presented a higher level of CCAT1 in cancer tissues than in para‐carcinoma tissues (Figure 1C). Then, we measured the expression level of CCAT1 in several LUAD cell lines. As shown in Figure 1D, the level of CCAT1 was significantly increased in LUAD cell lines. To determine the possible function and mechanism of CCAT1 in LUAD cells, we performed RNA pull‐down assay in A549 cell line and then analysed the retrieved proteins by Mass Spectrometry. As elucidated in Figure 1E and Table S1, a total of 66 proteins were identified to be binding partners with CCAT1. Then, we analysed the coding genes of these proteins by using the GO database (http://www.geneontology.org. Enrichment of these genes represents a measure of functions with significance, including ‘signal transduction’, ‘Energy pathways’ and ‘Metabolism’ (Figure 1F). Based on the analysis of GO, we selected fatty acid binding protein 5 (FABP5), which is critical factor for fatty acid (FA) metabolism and the mass spectrum peak of FABP5 was presented in Figure 1G. Moreover, protein‐protein interaction analysis revealed the interaction of FABP5 with FA metabolism‐related proteins such as peroxisome proliferator activated receptor (PPAR) and retinoid X receptor (RXR) (Figure 1H and Figure S1A). All these results indicated a possible regulation of CCAT1 on FA metabolism.

### 3.2 CCAT1 mediated the translocation of FABP5 into nucleus

**FIGURE 2:**
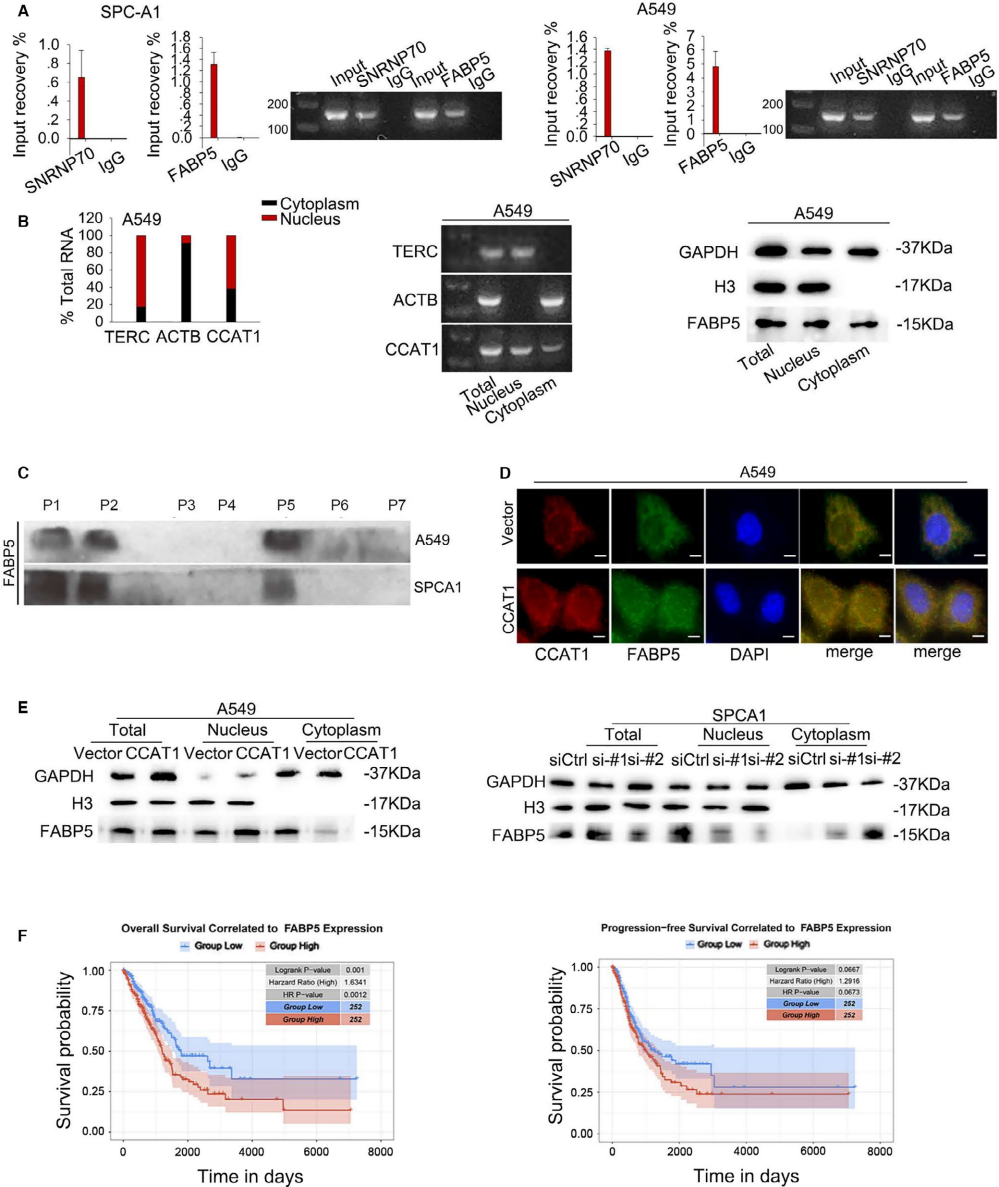
CCAT1 mediated the translocation of FABP5 into nucleus (A) RIP assay was carried out to confirm the interaction between CCAT1 and FABP5. Results analysed by Student's *t* test. (B) Subcellular fractionation was conducted to reveal the localization of CCAT1 and FABP5 protein. (C) Pull‐down assay validated the responsible binding domain of FABP5 on CCAT1. (D‐E) Expression level of FABP5 both in cytoplasm and nucleus was measured using immunofluorescence and Western blot assays. (F) Kaplan‐Meier analysis presented that high FABP5 level led to poor prognosis in LUAD patients. Three biological replicates each group for all experiments

To investigate the regulatory relationship between CCAT1 and FABP5, we analysed the interaction between CCAT1 and FABP5. The combination between CCAT1 and FABP5 was confirmed using RIP assays both in A549 and SPC‐A1 cell lines (Figure 2A). Since lncRNAs exhibited their functions dependent on their location, we then evaluated the subcellular localization of CCAT1 and FABP5. As shown in Figure 2B and Figure S1B, CCAT1 and FABP5 were located in both cytoplasm and nucleus. In order to further confirm the binding domain on CCAT1 for FABP5, we analysed the secondary structure of CCAT1 with the aid of bioinformatics tools (http://rna.tbi.univie.ac.at//cgi‐bin/RNAWebSuite/RNAalifold.cgi (Figure S1C) and fragmented it into seven sections. RNA pull‐down results showed an enrichment of FABP5 on the biotin labelled probes with P1, P2 and P5 (Figure 2C). Since the function of FABP5 is to translocate FA into nucleus, we transfected SPC‐A1 cells with CCAT1 expression vector and A549 cells with CCAT1 siRNA (Figure S2A) and measured the level of FABP5 both in cytoplasm and nucleus using immunofluorescence and western blot assays. As illustrated in Figure 2D and E, overexpressed CCAT1 enhanced the accumulation of FABP5 in nucleus, while knock‐down of CCAT1 led to an opposite result. Later, we analysed the correlation of FABP5 expression with the OS and DFS in lung cancer patients based on TCGA database. As presented in Figure 2F, patients with high FABP5 level tended to suffer from lower OS (Logrank *p* = 0.001, *n* (high) = 252, *n* (low) = 252), but DFS showed no statistical significance (Logrank *p* = 0.0667 *n* (high) = 252, *n* (low) = 252) in patients between FABP5 high and low expression groups. Results above indicated that CCAT1 could mediate the translocation of FABP5 into nucleus, and their interaction might be related to tumorigenesis of LUAD.

### 3.3 CCAT1 functioned synergistically with FABP5 to translocate FA into nucleus to induce PPAR‐RXR transcriptional complex and activate CD36, VEGF and PDK1

**FIGURE 3:**
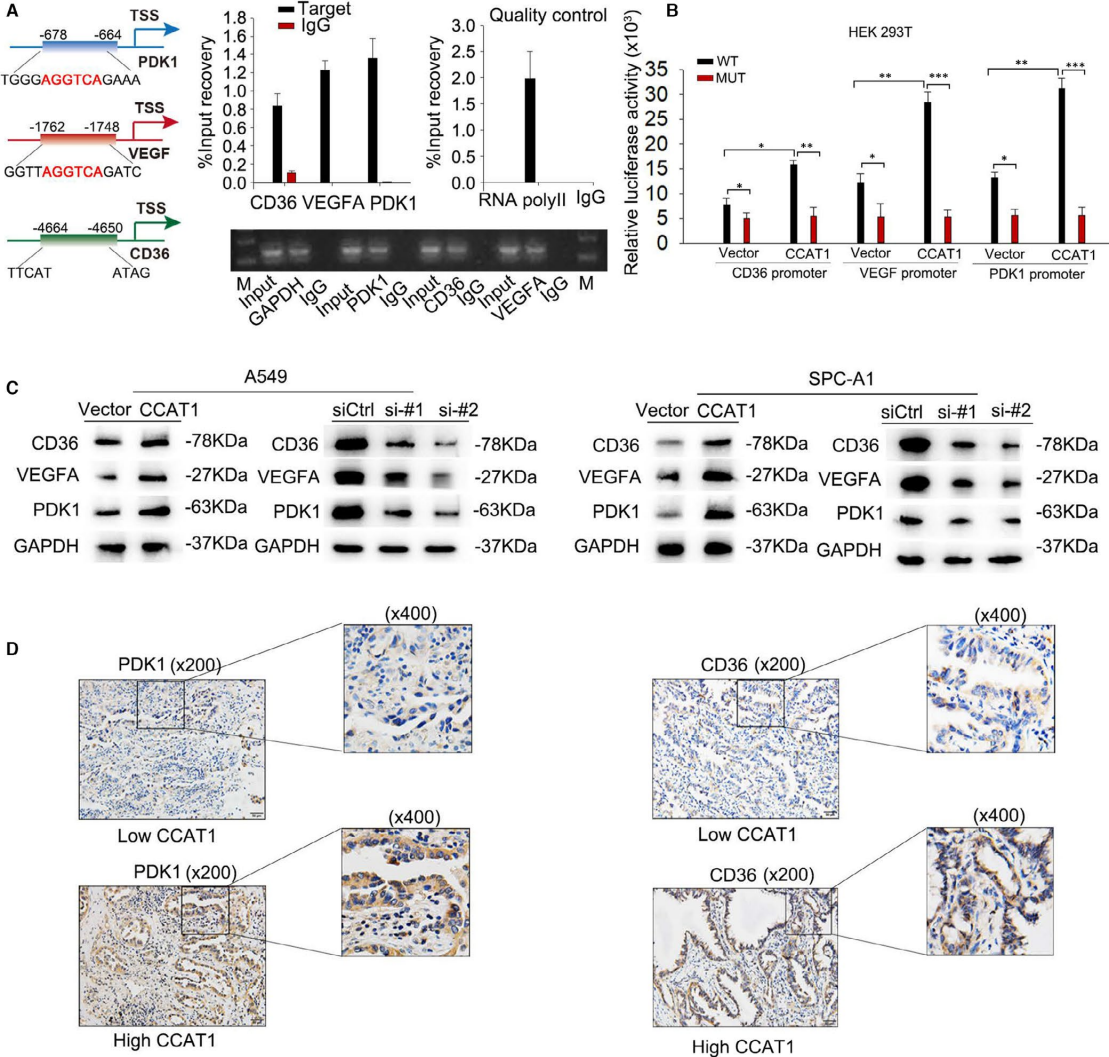
CCAT1 functioned synergistically with FABP5 to translocate FA into nucleus to induce PPAR‐RXR transcriptional complex and activate CD36, VEGF and PDK1 (A) Left: potential binding sites on promoter of the downstream genes with PPAR/RXR, including PDK1, VEGFA and CD36 were predicted. Right: ChIP assay was carried out to confirm the binding of PPAR and RXR on the promoter of PDK1, VEGFA and CD36. Results analysed by Student's *t* test. (B) Luciferase reporter assay was conducted to evaluate the effect of PPAR/RXR on the transcription of PDK1, VEGFA and CD36. Results analysed by two‐way ANOVA. (C) Western blot were used to test the protein levels of PDK1, VEGFA and CD36 under CCAT1 overexpression or silencing. (D) IHC analysis presented a high staining rate of PDK1, VEGFA and CD36 in tissues with high CCAT1 level. Three biological replicates each group for all experiments

It is well known that FA is essential for the activation of PPAR&RXR transcriptional complex and that PPAR&RXR mediated lipid metabolism.^18^ Therefore, we analysed the potential binding sites on promoters of the downstream genes for PPAR/RXR, including pyruvate dehydrogenase kinase 1 (PDK1), vascular endothelial growth factor A (VEGFA) and CD36 (Figure 3A left). ChIP assay validated the binding of the translational complex of PPAR and RXR on the promoter region of PDK1, VEGFA and CD36 (Figure 3A). Furtherly, luciferase reporter assay proved the activation of PPAR and RXR on the transcription of PDK1, VEGFA and CD36 (Figure 3B). Subsequently, we measured the expression of CD36, VEGFA and PDK1, in response to CCAT1 dysregulation. As revealed in Figure 3C, the protein levels of CD36, VEGFA and PDK1 were obviously increased in CCAT1‐overexpressed cells and decreased in CCAT1‐silenced cells. Moreover, we assessed the levels of CD36 and PDK1 in the LUAD tissues with high or low CCAT1 expression level. Consistently, LUAD tissues with high CCAT1 level presented a higher positive staining rates of CD36 and PDK1 than LUAD tissues with low CCAT1 level (Figure 3D). All these findings revealed that CCAT1 functioned synergistically with FABP5 to translocate FA into nucleus to induce PPAR&RXR transcriptional complex.

### 3.4 CCAT1 participated in reprogramming fatty acid metabolism

**FIGURE 4:**
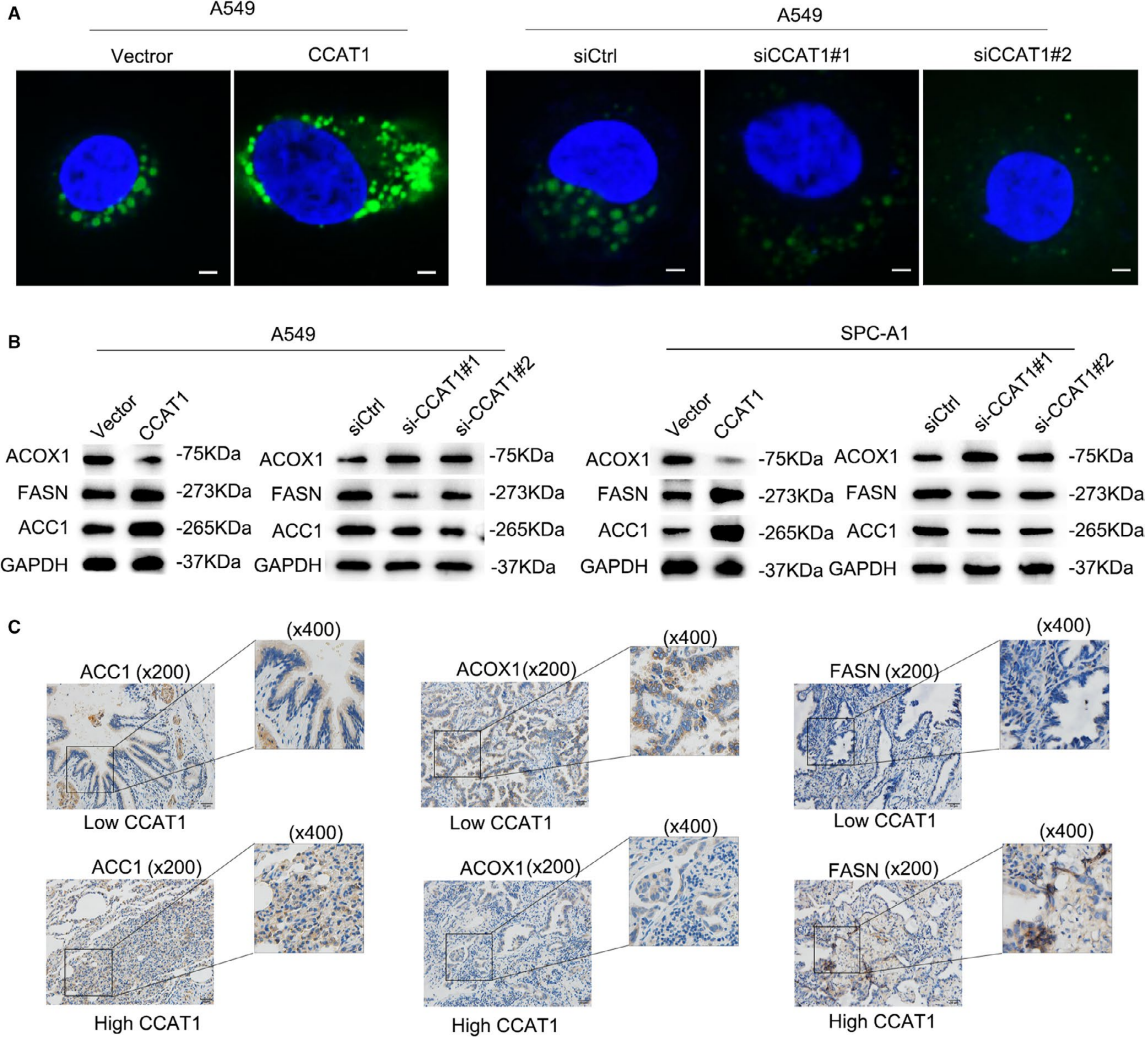
CCAT1 participated in reprogramming fatty acid metabolism (A) The lipophilic fluorescence dye BODIPY 493/503 (Invitrogen) was used to determine the level of neutral lipid accumulation in A549 cells. (B) Western blot was conducted to examine the protein levels of FASN, ACC1 and ACOX1 under the knock‐down or overexpression of CCAT1. (C) IHC staining was used to evaluate the levels of FASN, ACC1 and ACOX1 under the low or high expression of CCAT1 in LUAD tissues. Three biological replicates each group for all experiments

Fatty acid (FA), as a crucial energy provider for cells as well as the vital source for the generation of triglyceride, is closely related to the energy metabolism. Regulated by CCAT1 and PPARγ, the upregulation of CD36 is a vital transporter of fatty acid on cytomembrane that mediates the import of free fatty acid into cells.^19^, ^20^ FABP5, as one of the intracellular fatty acid‐binding proteins (FABPs), has been proposed to be a central regulator of FA metabolism. Increasing evidence has highlighted the pivotal role of FA metabolism in cancer metastasis, which has been reported to be regulated by lncRNAs via different mechanisms.^18^ Our findings above led us to believe that CCAT1 may be involved in reprogramming FA metabolism in LUAD. To assess the effects of CCAT1 on lipid content in LUAD cell lines, the levels of intracellular neutral lipids were detected by cellular staining with the lipophilic fluorescence dye BODIPY 493/503. The data presented that overexpression of CCAT1 increased the levels of neutral lipids in A549 cells, whereas silencing CCAT1 led to a lowered level of neutral lipids (Figure 4A). To demonstrate the role of CCAT1 in the deregulation on FA metabolism in LUAD, we examined the effects of CCAT1 on several other key FA metabolic enzymes, including fatty acid synthase (FASN), acetyl‐CoA carboxylase alpha (ACC1) and acyl‐CoA oxidase 1 (ACOX1) in LUAD cells. Consequently, significant down‐regulation of FASN and ACC1 and upregulation of ACOX1 protein levels were observed in comparison to the levels in control groups in A549 and SPC‐A1 cells after CCAT1 knock‐down (Figure 4B). And on the contrary, CCAT1 overexpression resulted in reverse impacts on the levels of these key FA metabolic enzymes in A549 and SPC‐A1 cells (Figure 4B). To provide further evidence, we measured the expression of FASN, ACC1 and ACOX1 in LUAD tissues with different CCAT1 levels using IHC staining, and obtained consistent results (Figure 4C). Thus, we concluded that CCAT1 promotes the reprogramming of FA metabolism in LUAD.

### 3.5 CCAT1 activated and stabilized mTOR pathway through competitively binding to USP49

**FIGURE 5:**
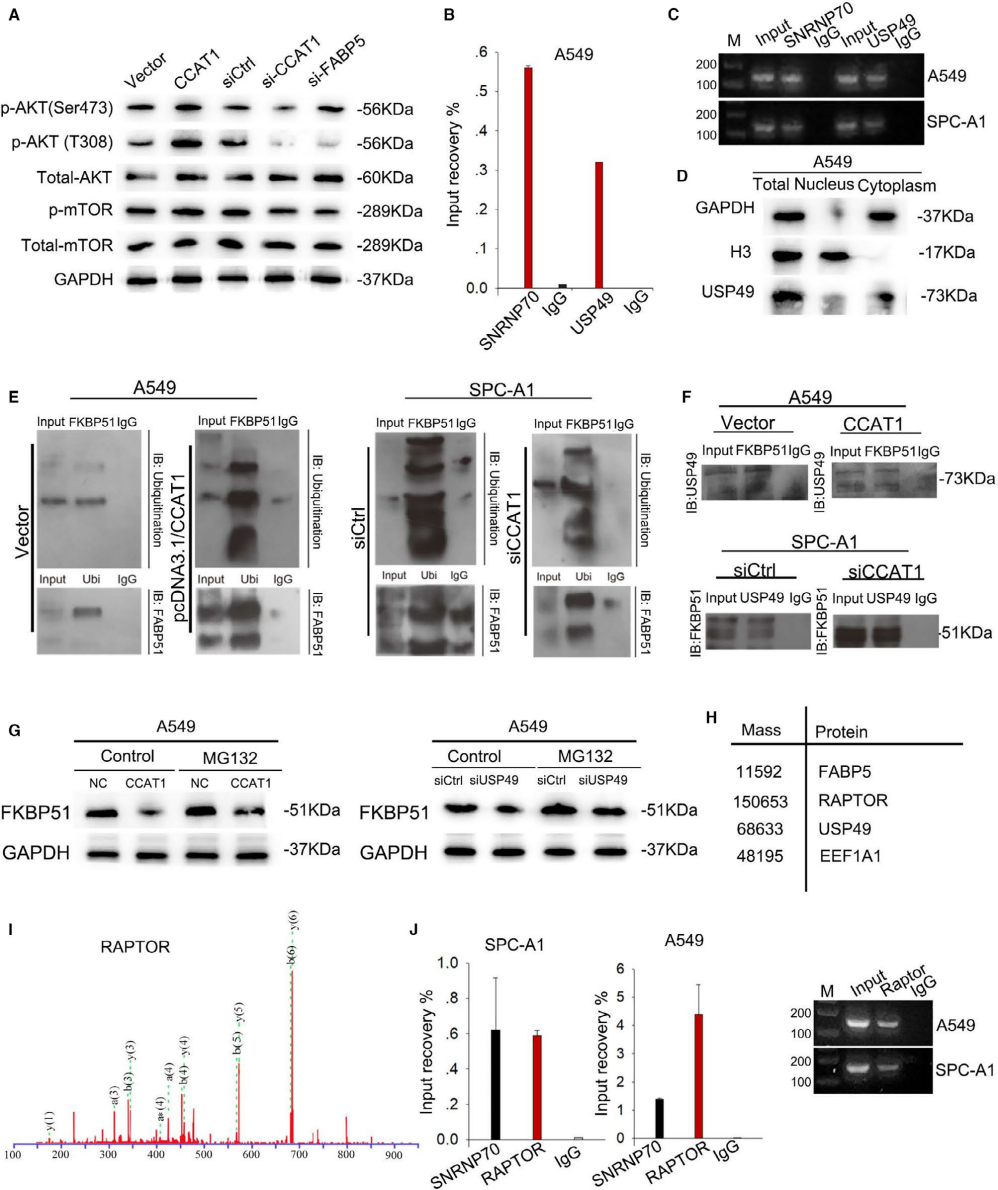
CCAT1 activated and stabilized mTOR pathway through competitively binding USP49 (A) Western blot was used to detect the level of p‐AKT (T308), p‐AKT (Ser473), total‐AKT, p‐mTOR and total‐mTOR in response to CCAT1 dysregulation. (B) RIP assay confirmed the interaction between CCAT1 and USP49. Results analysed by one‐way ANOVA. (C) qPCR results showed the enriched expression of CCAT1 on USP9‐binding complex. (D) Western blot revealed the predominant expression of USP49 in cytoplasm. (E) CoIP assay validated the effect of CCAT1 on FKBP51 ubiquitination. (F) CoIP assay was conducted to examine the interaction between FKBP51 and USP49 under the dysregulation of CCAT1. (G) Western blot was carried out to assess the effect of CCAT1 on the protein level of FKBP51. (H) Mass spectrometry analysis identified RAPTOR as a binding partner with CCAT1. (I) Mass spectrum peak of RAPTOR (J) RIP‐qPCR analysis was used to confirm the interaction between CCAT1 and RAPTOR. Results analysed by one‐way ANOVA. Three biological replicates each group for all experiments

Interestingly, the phosphoinositide 3‐kinase (PI3K)/AKT/mTOR signal pathway, one of the most commonly deregulated pathways in human cancers,^21^ has been verified to be modulated by PDK1 and VEGFA. Due to the findings that CCAT1 could up‐regulate PDK1 and VEGFA in an PPAR&RXR‐dependent manner, it is reasonable to believe that CCAT1 could modulate PI3K/AKT/mTOR pathway. To make confirmation, we found that the levels of p‐AKT (T308), p‐AKT (Ser473), and p‐mTOR were upregulated by CCAT1 overexpression and downregulated by CCAT1 silencing, with total‐AKT and total‐mTOR unchanged (Figure 5A). Specifically, PDK1 directly phosphorylated AKT on T308, but AKT phosphorylation on S473 required mTOC complex 2 (mTORC2), indicating that besides PDK1, there might be another pathway participated in the modulation of CCAT1 on PI3K/AKT/mTOR pathway.

By analysing the proteins binding with CCAT1, we found a deubiquitination enzyme ubiquitin specific peptidase 49 (USP49). Through collecting relevant literatures, reported by Huadong Pei et al. in 2010,^22^ we found that overexpression of FKBP51 resulted in a reduced phosphorylation of Akt at Ser473, but had no effect on the phosphorylation of Thr308. And in 2017, Kuntian Luo et al. demonstrated that USP49 deubiquitinated and stabilized FKBP51 so that the dephosphorylation of AKT at Ser473 was enhanced.^23^ Hence, we hypothesized that the interaction between CCAT1 and USP49 might modulate the stabilization of FKBP51. To prove our hypothesis, we first performed RIP assay and confirmed the combination between CCAT1 and USP49 in A549 cells (Figure 5B). As shown in Figure 5C, the qPCR products were in concordance with the data. Moreover, we found through Western blot that USP49 was mainly abundant in cytoplasm (Figure 5D and Figure S2B), which was consistent with the location of Ser473 phosphorylation of AKT. To explore the influence of CCAT1  on the ubiquitination of FKBP51, we transfected pcDNA3.1/CCAT1 or pcDNA3.1 vector into A549 and siCtrl or si‐CCAT1 into SPAC1 cells. We carried out CoIP assay with antibodies against FKBP51 or UBI and tested the UBI or FKBP51 enrichment in the precipitates. It was found that CCAT1 upregulation contributed to the ubiquitination of FKBP51 in A549 cell lines, while CCAT1 down‐regulation resulted in the deubiquitination of FKBP51 in SPC‐A1 cell lines (Figure 5E). Then, we analysed the combination between USP49 and FKBP51 in response to CCAT1 dysregulation by using co‐IP. As exhibited in Figure 5F, upregulation of CCAT1 blocked the combination between FKBP51 and USP49, whereas silenced CCAT1 promoted the combination between FKBP51 and USP49. Furthermore, we determined the effect of CCAT1/USP49 axis on the stabilization of FKBP51. As illustrated in Figure 5G, CCAT1 overexpression or USP49 knock‐down in A549 cells increased the protein level of FKBP51 but exhibited no effect on FKBP51 mRNA level (Figure S2C), and such effect was vanished by the addition of proteasome inhibitor MG132, suggesting that CCAT1 regulated FKBP51 levels in a proteasome‐dependent manner through competing binding with USP49.

Additionally, we identified the mTOR‐binding protein RAPTOR through mass spectrometry (Figure 5H) and protein‐protein interaction analysis (Figure S2D), and the mass spectrum peak of RAPTOR was presented in Figure 5I. And we confirmed through RIP‐qPCR the enrichment of CCAT1 in the Immunoprecipitate of RAPTOR antibody (Figure 5J), validating the binding between CCAT1 and RAPTOR. Collectively, these results combined with the uncovered regulation of CCAT1 on the activation of PDK1 translation and phosphorylation of AKT suggested the close association between CCAT1 and PI3K signalling. Therefore, we demonstrated that CCAT1 could stabilize PI3K pathway not only through stimulating PDK1 translation and AKT phosphorylation, but also through binding with RAPTOR. However, the underlying mechanism remains elusive.

### 3.6 CCAT1 enhanced malignant phenotypes of LUADs in vitro and vivo

**FIGURE 6:**
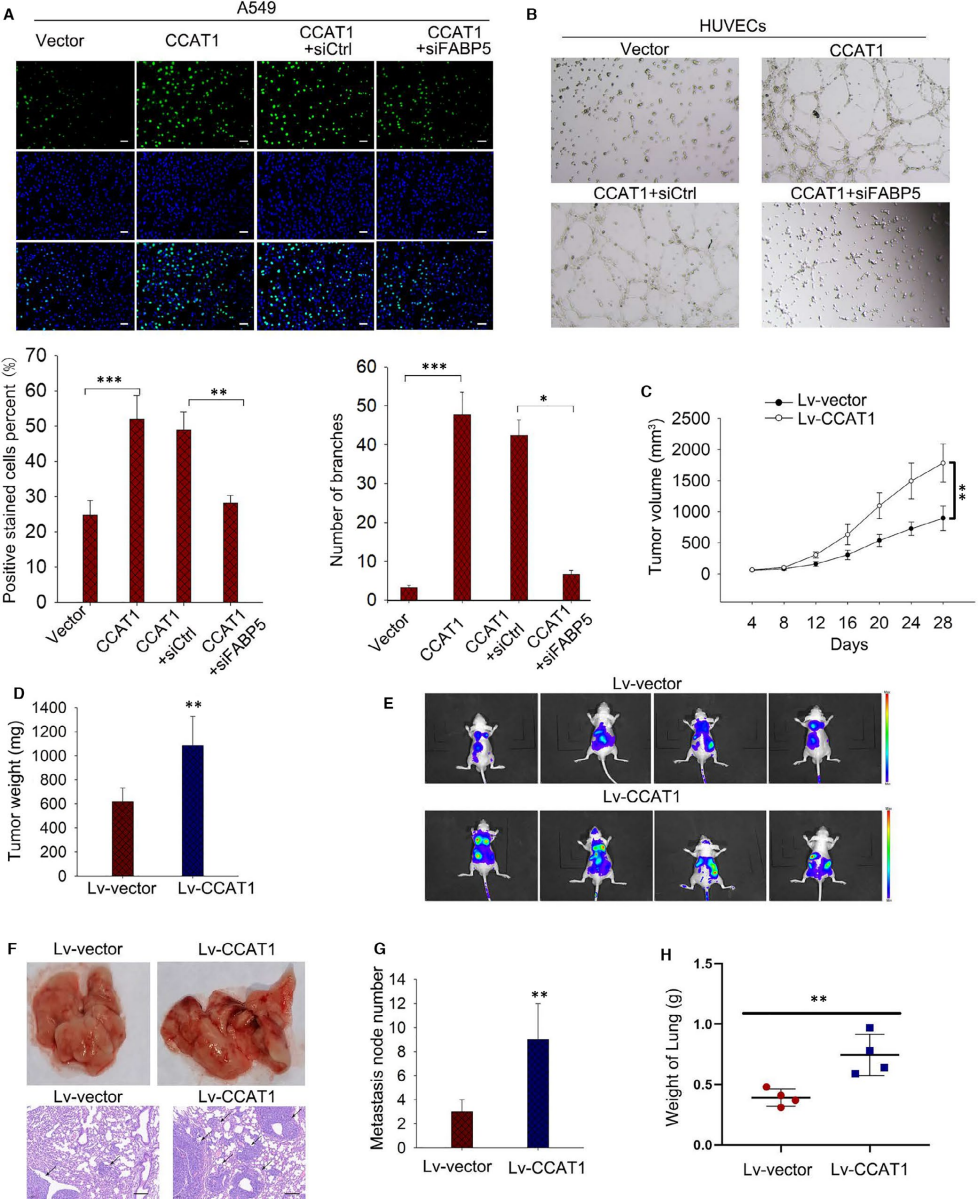
CCAT1 enhanced proliferation and angiogenesis in vitro and faciliated tumorigenesis and metastasis in vivo.(A) EdU assay were used to evaluate cell proliferation under CCAT1 overexpression or the co‐transfection of pcDNA3.1‐CCAT1 and siFABP5. (B) Tube formation assay was carried out to assess the angiogenesis ability of HUVECs under CCAT1 overexpression or the co‐transfection of pcDNA3.1‐CCAT1 and siFABP5. Results analysed by one‐way ANOVA. (C‐D) Xenograft assay was carried out with lv‐CCAT1 transfected mice with lv‐vector as control. Tumour volume and Tumour weight were measured. Results analysed by two‐way ANOVA. (E‐H) The effect of CCAT1 on number of metastatic nodes and lung weight were evaluated. Three biological replicates each group for all experiments

Since PI3K/AKT/mTOR signalling pathway plays a central role in regulating cell proliferation and angiogenesis, we evaluated the effect of CCAT1 on LUAD cell proliferation and angiogenesis. As shown in Figure 6A, forced expression of CCAT1 promoted A549 cell proliferation, and such phenomenon could be reversed by knockdown of FABP5. Results from co‐culture system revealed that overexpression of CCAT1 promoted the tube formation by HUVECs, and these effects were vanished when silencing FABP5 (Figure 6B). These results jointly indicated the involvement of CCAT1 and FAPB5 in the promotion of malignant phenotypes in LUAD. To verify the observations obtained in vitro, we constructed A549 cells with stable lv‐CCAT1 expression vector, while cells transfected with lv‐vector were used as negative controls. Then, cells were injected into nude mice to assess the effect of CCAT1 on LUAD tumorigenesis in vivo. It was discovered that A549 cells stably transfected with lv‐CCAT1 generated tumours with faster speed than control cells in mice (Figure 6C‐D). Moreover, a luciferase xenograft mouse model was established to determine whether CCAT1 affected tumour metastasis growth in vivo. A549 cells stably transfected with lv‐CCAT1 were subcutaneously injected into the tail veins of nude mice. As shown in Figure 6E‐H, ectopic CCAT1 expression increased the number of metastatic nodes and lung weight compared with the control group. Therefore, the in vivo investigations complemented the in vitro functional experiment results involving CCAT1.

## 4 DISCUSSION

CCAT1 was initially reported in colorectal cancer as a diagnostic biomarker.^24^, ^25^ Later, with accumulating deep researches, the role of CCAT1 in cancers has been increasingly identified, such as in oesophageal cancer and gallbladder carcinoma.^17^, ^26^ The upregulation of CCAT1 in lung adenocarcinoma has been identified, but its regulatory mechanism remains poorly understood. Fatty acid is known to be an energy provider for bodies, playing a central role in tumorigenesis and tumour progression.^27^ Its biological activities require the aid of fatty epidermal acid binding proteins, which exert important regulatory roles in various metabolism‐related diseases.^28^ However, its association with cancers, especially with lung adenocarcinoma, remains in the dark. Present study discovered that CCAT1 was involved in the FA metabolism through binding with FABP5 so as to regulate downstream gene expression, which therefore promoted LUAD via PI3K/AKT/mTOR signalling.

The reprogramming of cellular energy metabolism, as emerging hallmarks of cancer, supports the infinite multiplication and metastatic progression of cancer cells.^29^ Multiple molecular regulatory mechanisms, intrinsic and extrinsic, converge to alter core cellular metabolism. Metabolic changes are a common feature of cancerous tissues. The most commonly observed metabolic phenotype in tumour cells is the Warburg effect, which is characterized with an increased uptake of glucose and the switch from ATP generation through oxidative phosphorylation to ATP generation through glycolysis.^30^ Emerging documents indicate that the Warburg effect may favour the early stages of cancer progression. Fatty acid (FA), one of the major building blocks for energy metabolism, represents the essential component of the signal transduction network of biological membranes. FA metabolism provides cancer cells a selective advantage for the metastatic process.^31^ The intracellular fatty acid‐binding proteins (FABPs), as indispensable carriers for FA uptake and transporting, are abundantly expressed in almost all tissues and proposed to be vital central regulators of FA metabolism.^32^ FABP5, a small (~15 kD) member of the cytoplasmic FABP family, exhibits high affinity with FAs. Accumulating convincing evidence has revealed that FABP5 exhibits a crucial role in tumorigenesis, as well as the induction of EMT.^33^, ^34^

FABP5/PPARγ pathway is recognized to be important for the development of LUAD. PPARγ is a transcription factor activated by its receptor, regulating downstream gene expression under the formation of transcriptional complex by FA and RXR. Through RNA pull‐down with CCAT1 biotin‐labelled probes followed by mass spectrometry analysis, we identified the interaction between CCAT1 and FABP5, which was later confirmed by RIP‐qPCR assay. Additionally, though documentary research, we found that FA is not only an important source for body energy, but also a stimulator of downstream gene translation depending on PPARγ. Through IF and subcellular fractionation, we validated that CCAT1 could mediate the translocation of FABP5 into nucleus.

It has been known that CD36 is an important receptor of FA on cytomembrane which mediates FA into cells. PDK1 is the pivotal protein in PI3K signalling activating the phosphorylation of AKT (T308), which is acknowledged to be closely associated with tumour progression. And VEGFA is an angiogenesis‐related protein which relates to the activation of PI3K signalling. Present study verified through bioinformatics prediction and ChIP the binding of PPARγ and RXR to the promoters of CD36, PDK1 and VEGFA. And luciferase reporter assay presented the promotion of PPARγ/RXR on the luciferase activity of their promoters, indicating that PPARγ/RXR promoted the expression of these genes through activating their transcription. Also, in vitro assay proved that CCAT1 overexpression contributed to the upregulation of the three genes.

The phosphoinositide 3‐kinase (PI3K)/AKT/mTOR signal pathway is identified as one of the most commonly deregulated pathways in human cancers.^21^ Genetic aberrations that modulate this pathway, like inactivation of PTEN or activated mutations of PIK3CA, have been documented in virtually all epithelial tumours.^35^ The 3‐phosphoinositide‐dependent protein kinase‐1 (PDK1), whose activation is known to be a consequence of the accumulation of phosphatidylinositol‐3,4,5‐trisphosphate (PIP3), is considered as a critical component of the PI3K pathway. PDK1 is a master regulator of AGC kinase members playing multiple roles in various physiologic processes, including metabolism, growth, proliferation and survival.^36^ It has been known that PDK1 is a key protein in PI3K signalling promoting the phosphorylation of T308 on AKT, which is closely associated with the development of tumours and plays an important role in multiple signalling for the tumorigenesis.

As reported by Huadong Pei et al. in 2010,^22^ overexpression of FKBP51 resulted in a reduced phosphorylation of Akt at Ser473, but had no effect on the phosphorylation of Thr308. And in 2017, Kuntian Luo et al. demonstrated that USP49 deubiquitinated and stabilized FKBP51 to enhance the dephosphorylation of AKT at Ser473.^23^ In current study, we identified USP49 in the retrieved proteins by pull‐down, and confirmed that it is a regulator of the ubiquitination of FKBP51. FKBP51 could inhibit PI3K pathway through phosphorylating AKT S473 via suppressing mTOR2. In our study, CCAT1 was found to block the deubiquitination of FKBP51 by recruiting USP49, which maintained the activity of AKT. And another protein identified by mass spectrometry RAPTOR help us validated that CCAT1 positively regulated PI3K signalling. RIP assay proved the interaction between CCAT1 and RAPTOR. However, through what mechanism CCAT1 regulated RAPTOR and mTOR1 remains to be uncovered. Furthermore, for the influence of CCAT1 on fat acid metabolism, we verified that CCAT1 silencing suppressed the expression of fat acid metabolism‐related proteins such as FASN, ACC1 and ACOX1.^37^ Previously, we also found that CCAT1 played an essential role in regulating the drug resistance of LUAD, so we speculated that it might function through PI3K signalling, and we will deepen this study in the future.

**FIGURE 7:**
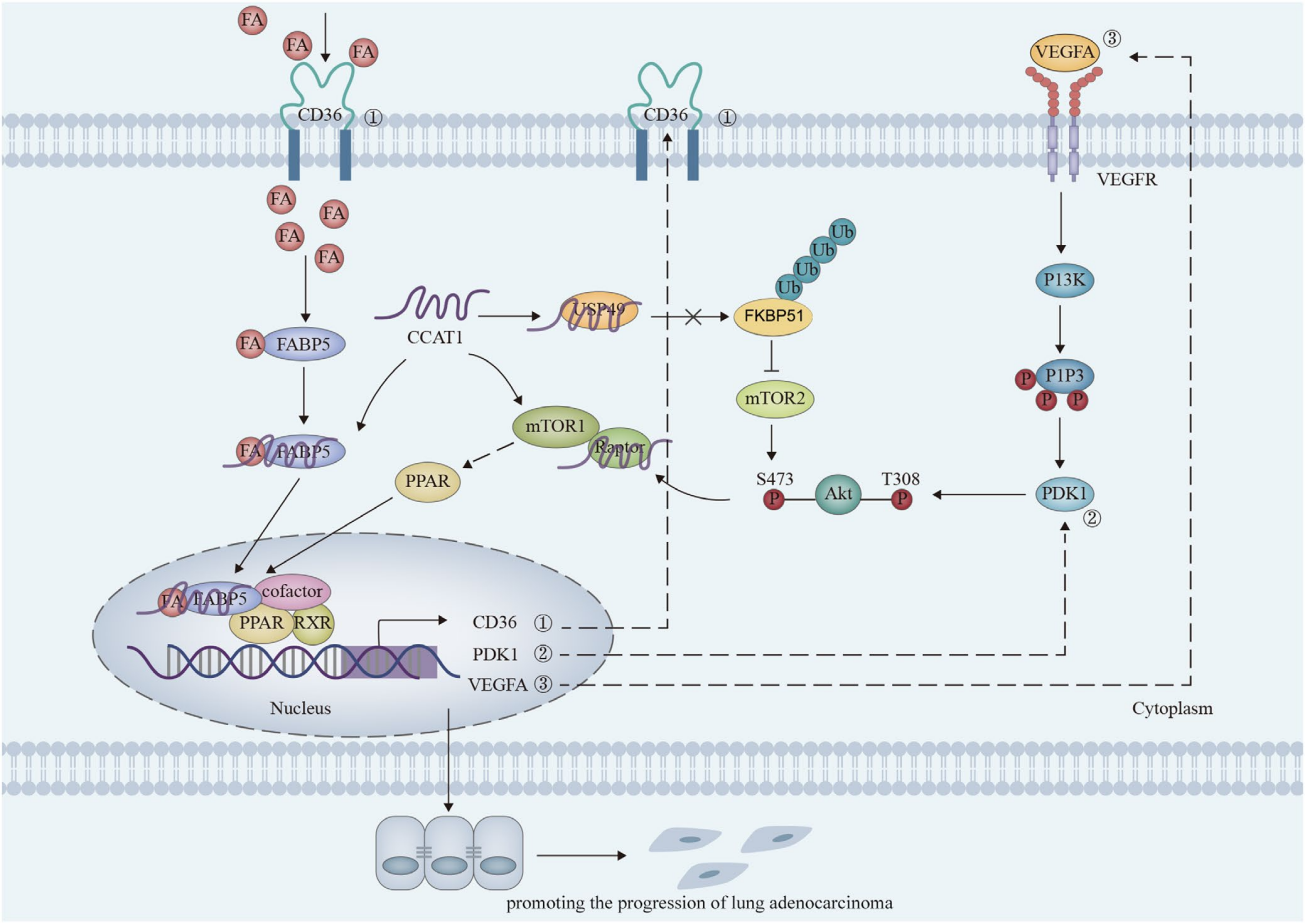
Science schematic of current work: CCAT1 participates in FA metabolism through regulating the translocation of FABP5 into nucleus to stabilize PI3K/AKT/mTOR signalling, therefore promoting the progression of lung adenocarcinoma

In conclusion, the promotion of CCAT1 on fat acid metabolism not only provides necessary energies for the biological processes of cancer cells, but also contributes significantly to angiogenesis and activation of tumour‐related signalling pathways (Figure 7). These findings provide valuable diagnostic and prognostic targets for LUAD patients, and unfold a new outlook for the research on the mechanism underlying LUAD.

## CONFLICT OF INTEREST

The authors confirm that there are no conflicts of interest.

## AUTHOR CONTRIBUTIONS

**Jing Chen:** Conceptualization (lead); Project administration (lead); Supervision (lead); Writing‐original draft (lead); Writing‐review & editing (lead). **Yaser Alduais:** Data curation (lead); Writing‐original draft (supporting). **Kai Zhang:** Formal analysis (lead); Investigation (lead); Methodology (lead). **Xiaoli Zhu:** Writing‐original draft (supporting); Writing‐review & editing (supporting). **Baoan Chen:** Project administration (equal); Writing‐original draft (supporting); Writing‐review & editing (supporting).

## Supporting information

Fig S1

Fig S2

Table S1

## Data Availability

All data obtained from this work have been presented within the manuscript and its related files.
