# Supplementary figures and images for: CCAT1/FABP5 promotes tumour progression through mediating fatty acid metabolism and stabilizing PI3K/AKT/mTOR signalling in lung adenocarcinoma
Source: J Cell Mol Med. 2021 Aug 25;25(19):9199–213. doi: 10.1111/jcmm.16815 (PMC8500980; doi:10.1111/jcmm.16815)

A

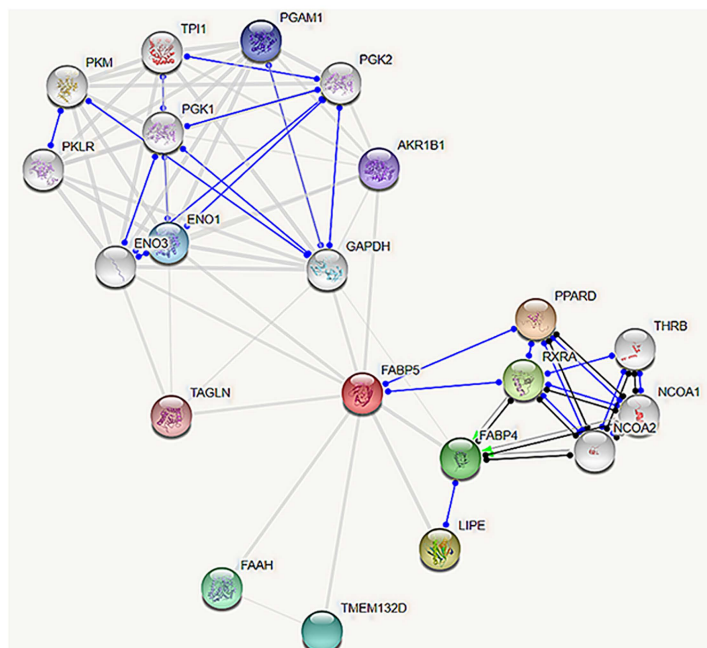

B

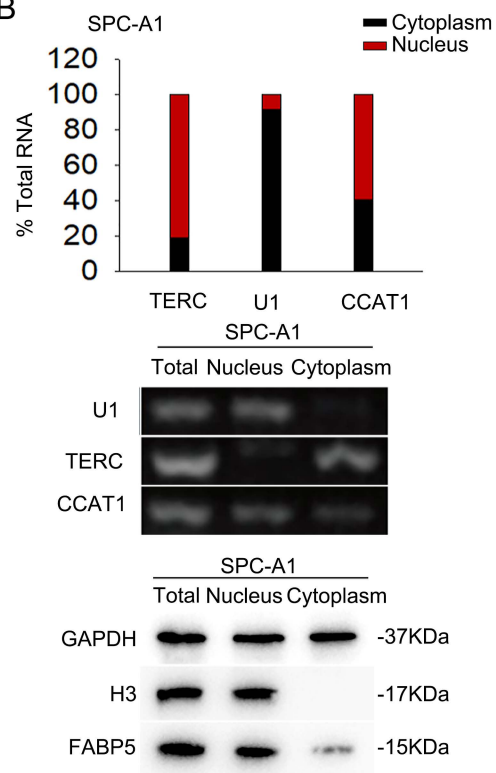

C

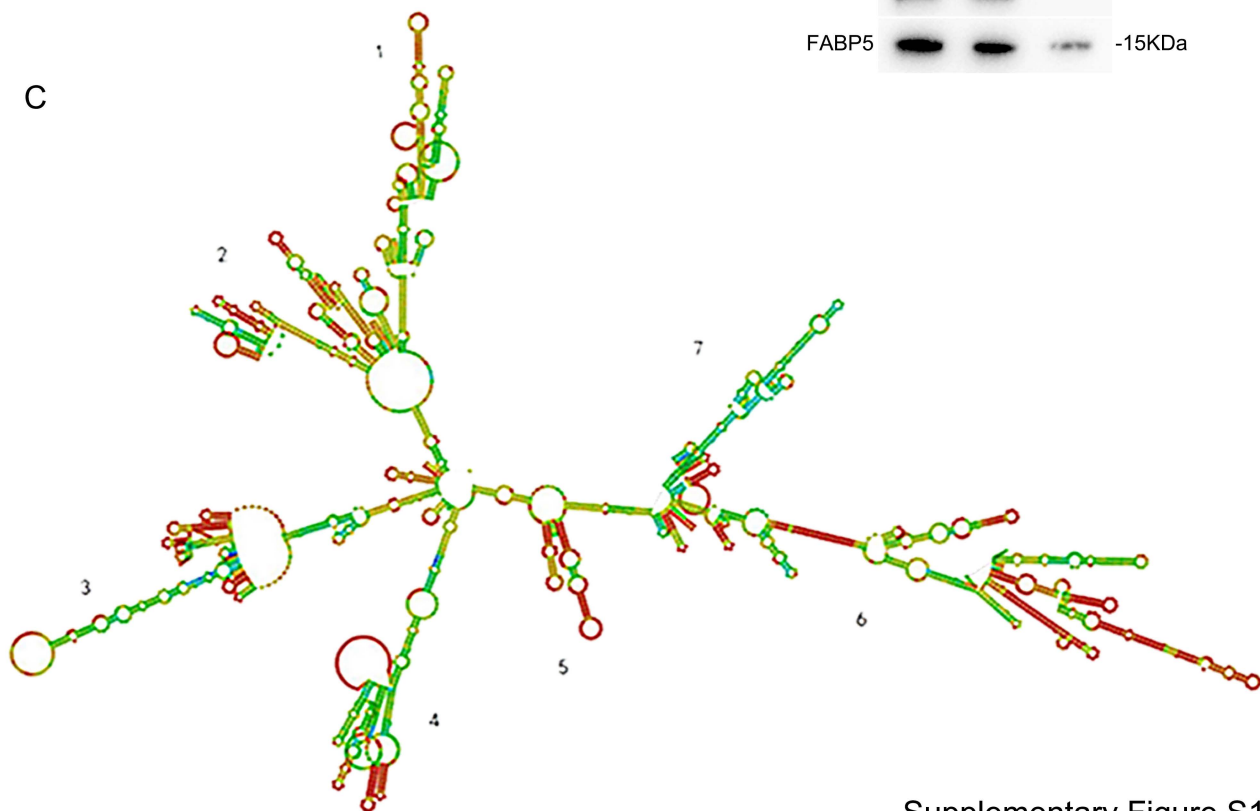

Supplementary Figure S1

Supplement: Supplementary file 1 — Fig S1 [file JCMM-25-9199-s001.pdf]

**A**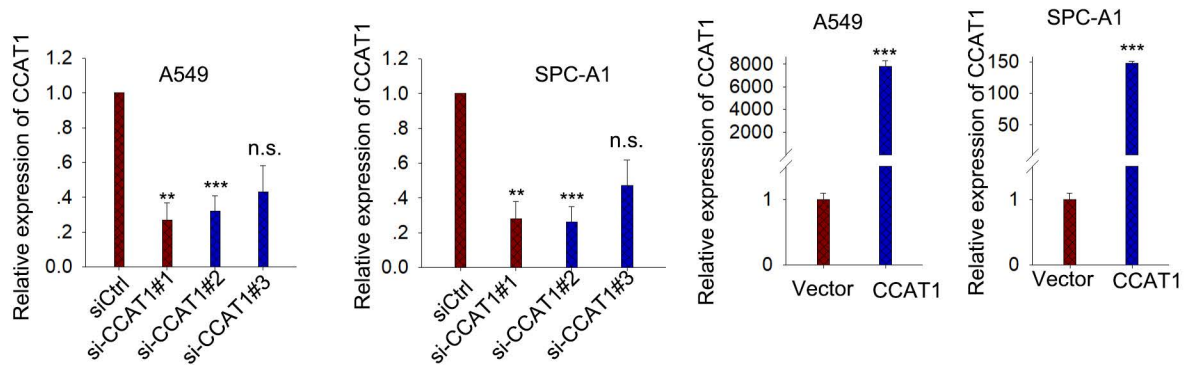**B**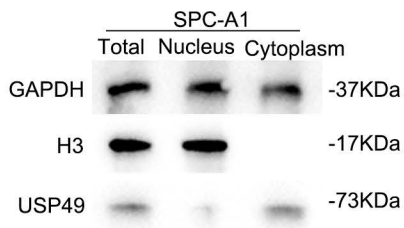**C**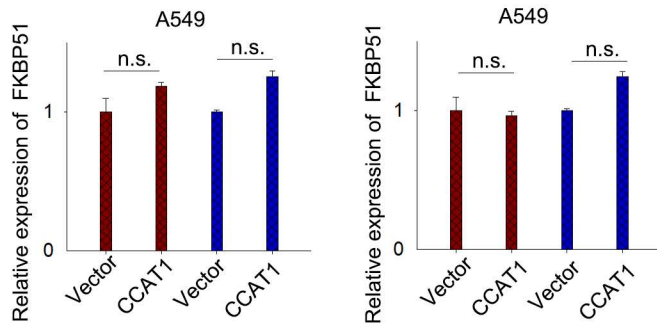**D**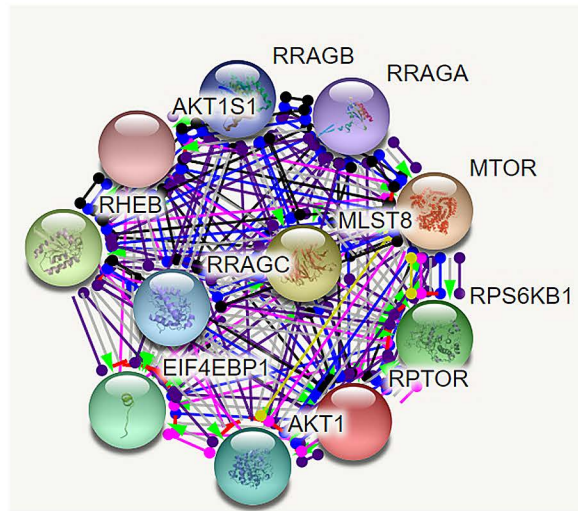

Supplementary Figure S2

Supplement: Supplementary file 2 — Fig S2 [file JCMM-25-9199-s002.pdf]
